# Reduction of claustrophobia during magnetic resonance imaging: methods and design of the "CLAUSTRO" randomized controlled trial

**DOI:** 10.1186/1471-2342-11-4

**Published:** 2011-02-10

**Authors:** Judith Enders, Elke Zimmermann, Matthias Rief, Peter Martus, Randolf Klingebiel, Patrick Asbach, Christian Klessen, Gerd Diederichs, Thomas Bengner, Ulf Teichgräber, Bernd Hamm, Marc Dewey

**Affiliations:** 1 Departments of Radiology, Charité, Medical School, Humboldt Universität zu Berlin and Freie Universität Berlin, Germany; 2 Departments of Biostatistics and Clinical Epidemiology, Charité, Medical School, Humboldt Universität zu Berlin and Freie Universität Berlin, Germany; 3 Divisions of Neuroradiology, Charité, Medical School, Humboldt Universität zu Berlin and Freie Universität Berlin, Germany; 4 Departments of Clinical Psychology, Charité, Medical School, Humboldt Universität zu Berlin and Freie Universität Berlin, Germany

## Abstract

**Background:**

Magnetic resonance (MR) imaging has been described as the most important medical innovation in the last 25 years. Over 80 million MR procedures are now performed each year and on average 2.3% (95% confidence interval: 2.0 to 2.5%) of all patients scheduled for MR imaging suffer from claustrophobia. Thus, prevention of MR imaging by claustrophobia is a common problem and approximately 2,000,000 MR procedures worldwide cannot be completed due to this situation. Patients with claustrophobic anxiety are more likely to be frightened and experience a feeling of confinement or being closed in during MR imaging. In these patients, conscious sedation and additional sequences (after sedation) may be necessary to complete the examinations. Further improvements in MR design appear to be essential to alleviate this situation and broaden the applicability of MR imaging. A more open scanner configuration might help reduce claustrophobic reactions while maintaining image quality and diagnostic accuracy.

**Methods/Design:**

We propose to analyze the rate of claustrophobic reactions, clinical utility, image quality, patient acceptance, and cost-effectiveness of an open MR scanner in a randomized comparison with a recently designed short-bore but closed scanner with 97% noise reduction. The primary aim of this study is thus to determine whether an open MR scanner can reduce claustrophobic reactions, thereby enabling more examinations of claustrophobic patients without incurring the safety issues associated with conscious sedation. In this manuscript we detail the methods and design of the prospective "CLAUSTRO" trial.

**Discussion:**

This randomized controlled trial will be the first direct comparison of open vertical and closed short-bore MR systems in regards to claustrophobia and image quality as well as diagnostic utility.

**Trial Registration:**

ClinicalTrials.gov: NCT00715806

## Background

Magnetic resonance (MR) imaging has been described as the most important medical innovation in the last 25 years [1]. There has been an enormous increase in the use of this modality in the clinical setting. Over 80 million MR procedures are now performed each year worldwide. For an MR scan, patients typically have to be placed in a long, narrow tube [2]. Thus, claustrophobia preventing MR imaging is a common problem. Between 1% and 15% of all patients scheduled for MR imaging suffer from claustrophobia and cannot be imaged, or they require sedation to complete the scan (mean: 2.3%; 95% confidence interval: 2.0% to 2.5%) [3]. Thus it can be estimated that worldwide approximately 2,000,000 MR procedures cannot be performed or are prematurely terminated due to claustrophobia. At an average cost of € 500 per MR imaging, this is equal to a loss of productivity of € 1 billion, which is an important financial loss for the health care system. Therefore, claustrophobia is not only a common problem that prevents many patients from benefiting from the findings obtained with MR imaging but also represents an important socioeconomic issue for the health care system.

Patients with claustrophobic anxiety are more likely to be frightened and experience a feeling of confinement or being closed in during MR imaging [4,5]. Those anxious patients have been reported to experience claustrophobia in up to 35% of all cases [6]. In these patients conscious sedation and additional sequences (after sedation) may be necessary to complete the examinations. This situation involves significant risks of adverse events [7-9] and is associated with extra costs because it reduces workflow, limits patient acceptance, and wastes valuable scanning time. Improving comfort during MR imaging (mainly reduction of noise and sensations of confinement by a fairly panoramic view) appears to be essential to avoid claustrophobic reactions [10,11]. The positive effect of noise reduction and improved patient-centered design (short and wide bore) has recently been shown in a large cohort study in over 55,000 patients using a newly designed closed MR unit (with 97% noise reduction and cylindrical CT-like appearance) with 1.5-T field and up to 45 mT/m gradient strength [3]. However, even on such recent scanners, claustrophobic patients cannot undergo MR imaging without potentially life-threatening sedation.

A more open vertical scanner configuration might be an alternative, but until recently such systems operated at rather low field strengths (0.2 T), resulting in poor image quality especially in imaging of the brain [12]. Thus, recently introduced open MR scanners with an all-around view and both high field (1.0 T) and high gradient strength (up to 26 mT/m) might be a valuable clinical alternative to further reduce claustrophobia while maintaining diagnostic image quality and diagnostic accuracy [13,14]. The more all-around view from the inside of open MR scanners is likely to reduce the incidence of claustrophobia. However, new closed MR scanners with patient-centered design (short and wide bore) and noise reduction have also been shown to greatly reduce claustrophobic reactions by a factor of up to 3 [3]. Nevertheless, no direct comparison of these two scanner types in regards to claustrophobia and image quality as well as diagnostic utility has been performed thus far. Therefore, it is unclear whether an open vertical or a closed short-bore MR design is more effective in reducing claustrophobia that prevents MR imaging. Moreover, by itself the design of open MR scanners cannot guarantee a reduction in claustrophobia or an improvement in patient outcome. The most decisive evidence for judging the efficacy of diagnostic tests comes from randomized comparisons in which the controlled design eliminates the biases of observational studies [15,16]. Randomized trials are needed to define the clinical role of such open MR scanners in patients with claustrophobic anxiety. Also no study has addressed which specific scanner design patients would prefer for imaging. Therefore, this study is designed to determine whether an open vertical or a closed short-bore MR design is more effective in alleviating claustrophobic anxiety preventing MR imaging and which further improvements might be necessary to reduce claustrophobia during MR imaging.

## Methods/Design

### Primary and secondary objectives

We propose to analyze the rate of claustrophobic reactions and clinical utility of an open MR scanner in a randomized comparison with a recently designed short-bore but closed scanner with 97% noise reduction. This trial will be the first to appraise the potential for claustrophobia reduction and clinical relevance of open MR scanners in claustrophobic patients with a clinical indication for MR imaging. Furthermore, this trial will analyze and compare the cost-effectiveness of the two MR scanners, which is important in view of the enormous annual loss of healthcare productivity due to claustrophobia during MR imaging. Also, patient preferences and image quality will be analyzed. Thus, this randomized trial may have the potential to influence both the clinical and economical utilization of MR imaging. If an open MR scanner can be shown to reduce claustrophobia in a randomized comparison it might be justified to recommend this approach for routine clinical application in certain high-risk anxiety patients or in certain imaging centers.

**Figure 1:**
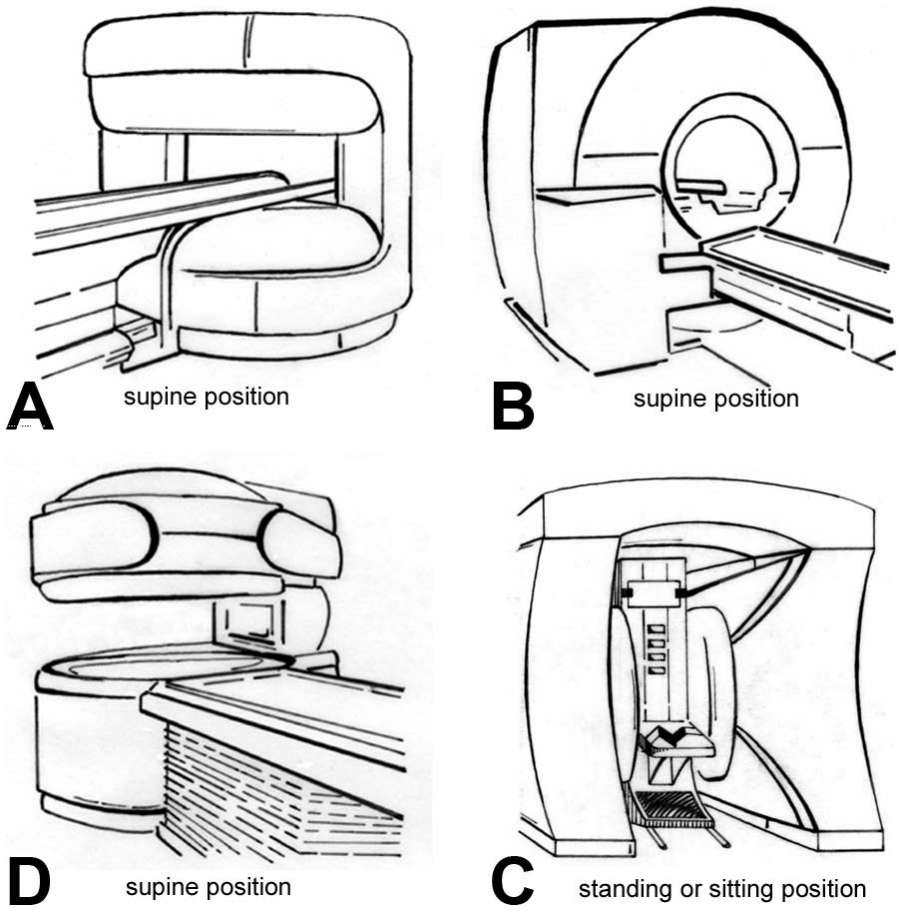
**Drawings of four MR scanners which will be used in the custom-made questionnaire to assess which specific MR scanner design is evaluated to be the most attractive**. Patients will be asked to rank the four MR scanner types according to their preferences. First, this assessment will be done assuming equal diagnostic utility of the MR scanners, and second, assuming for scanner **A **good, for scanner **B **very good, for scanner **C **moderate and for scanner **D **adequate diagnostic utility. **Panel A **shows the open MR scanner with a vertical magnetic field design and 1-T field strength (Panorama, Philips). **Panel B **shows the closed MR scanner with a short- and wide-bore design and 1.5-T field strength (Magnetom Avanto, Siemens). **Panel C **shows an open MR scanner with a one column design and a 0.4-T vertical magnetic field. **Panel D **shows a MR scanner with a 0.6-T horizontal magnetic field and a top/front-open design which allows upright positioning of the patient, while patients have to be examined in supine position on the scanners **A-C**. This information will allow defining which further improvements by the vendors might be necessary to reduce claustrophobic reactions during MR imaging.

The primary aim of this study is thus to determine the ability of an open MR scanner to reduce claustrophobic reactions, thereby expanding the use of MRI in patients with claustrophobic anxiety. To achieve this, claustrophobic reactions (while entering the scanner room or during the MR procedure) that prevent MR imaging without sedation will be compared in the two randomized study groups. Thus, the question to be answered by this project is whether an open vertical or a short-bore MR design will alleviate claustrophobia that prevents MR imaging to a greater extent. The main secondary aims are the correlation of patients' scores in several validated psychological self-report questionnaires with the occurrence of claustrophobic events, the duration of MR imaging in the two groups, and to systematically analyze if and what further design improvements might be necessary to reduce claustrophobia during MR imaging in the future. Therefore, patient preferences concerning MR scanner design will be determined (Figure 1, Table 1). These insights are important to be able to define which further improvements by vendors might be necessary to reduce claustrophobic reactions during MR imaging.

**Table 1:** Summary of the items addressed in the custom-made questionnaire to assess satisfaction with the MR scan and preferences concerning MR scanner design

Item	Range
Preparation and information	Excellent/Good/Satisfactory/Fair/Poor
Concern	Not at all/Very little/Little/High/Very high
Comfort	Excellent/Good/Satisfactory/Fair/Poor
Feeling helpless/embarrassed	Not at all/Little/Moderate/Strong/Very strong
Anxiety	Horizontal visual analogue scale of 10 cm (0-100)
Noise	Horizontal visual analogue scale of 10 cm (0-100)
Pain	Horizontal visual analogue scale of 10 cm (0-100)
Complications	Yes/No/If yes, which complications
Reasons for premature termination (if applicable)	Free text
Reasons for successful completion (if applicable)	Free text
Willingness to undergo future examinations on the MR scanner	Yes/No/Maybe
General satisfaction with the MR scan	Excellent/Good/Satisfactory/Fair/Poor
**Summary**	
Advantages/Disadvantages of the respective MR scanner	Free text
Ranking of four MR scanners of different design assuming equal diagnostic utility (Figure 1)	1 to 4
Ranking of four MR scanners assuming different diagnostic utility (Figure 1)	1 to 4
Ranking of the two MR scanners used in the study (Figure 2)	1 to 2
Advantages/Disadvantages of the two MR scanners used in the study	Free text

Further secondary aims are the influence of MR imaging results on clinical management in the two groups, cost-effectiveness of the two scanners, image quality achieved in claustrophobic patients using the two scanners, patient acceptance and preference in regards to the diagnostic procedure in the two randomized study groups, and the development of patients' anxiety during 4 to 6 weeks and 7 months after MR imaging.

### Design

**Figure 2:**
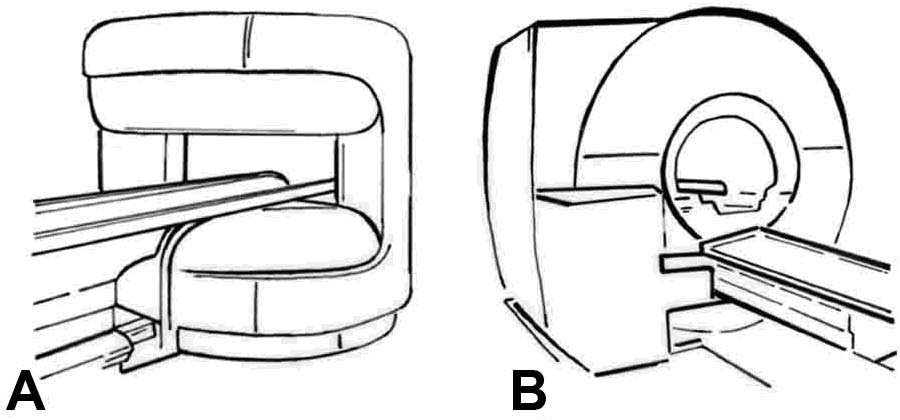
**Drawings of the two MR scanners to which patients will be randomized in the study**. **Panel A **shows the open MR scanner with a vertical magnetic field with 1.0-T field strength (Panorama, Philips), up to 26 mT/m gradient strength, acoustic scanner noise of maximum 150 dB(A), and a 0.45 m high and 1.6 m wide patient aperture (0.7 m wide patient table). **Panel B **shows the closed MR scanner with 1.5-T field strength, up to 45 mT/m gradient strength, 97% noise reduction to below 99 dB(A), and a conical shaped wide (0.6 m) and short (1.5 m) bore resembling the gantry of a computed tomography scanner, which has been shown to reduce claustrophobia by a factor of 3 (Magnetom Avanto, Siemens).

**Figure 3:**
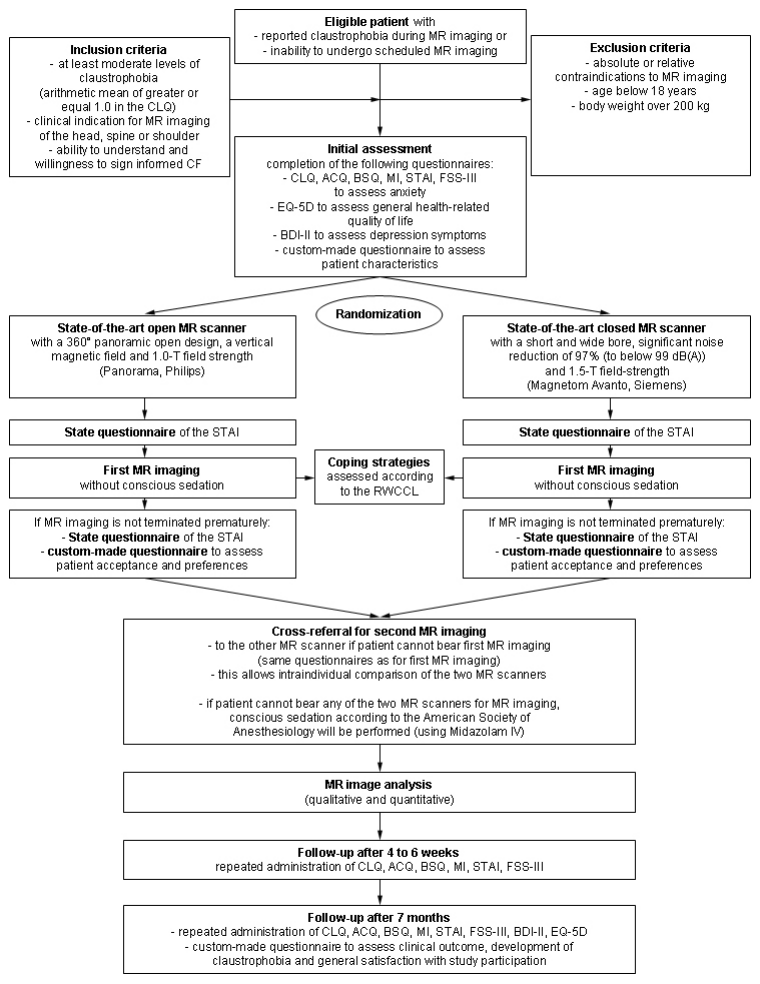
**Chart of CLAUSTRO study design. The diagram depicts the randomization procedure, patient flow, and data analysis**. **Abbreviations: **AKV = Fragebogen zu körperbezogenen Ängsten, Kognitionen und Vermeidung [19]. BDI-II = Beck Depression Inventory II [24]. CF = Consent Form. CLQ = Claustrophobia Questionnaire [21]. FSS-III = Fear Survey Schedule III [20]. RWCCL = Revised Ways of Coping Checklist [29]. STAI = State-Trait Anxiety Inventory [18].

This is a randomized controlled trial of patients with reported claustrophobia during prior MR imaging or with the inability to undergo MR imaging in conventional scanners. Eligible patients will be randomly assigned to one of the following study groups. 1) The open MR scanner: imaging will be performed in a state-of-the-art scanner with a vertical magnetic field and 360° open design and 1-T field strength (Panorama, Philips, Figure 2A) [14], and 2) A state-of-the-art MR scanner (control group) without an open design but significant noise reduction of 97% (to below 99 dB(A)) and patient-centered design (short and wide bore) with 1.5-T field strength, which has already been shown to reduce claustrophobia by a factor of 3 compared with conventional MR scanners (Magnetom Avanto, Siemens, Figure 2B) [3]. The study design is shown in Figure 3.

In order to improve patient acceptance, patients who are assigned to imaging in one MR scanner but cannot bear this procedure due to claustrophobia will not be sedated (with conscious sedation according to the standards of the American Society of Anesthesiology) [17], but will be cross-referred to imaging in the other scanner in order to preclude adverse events associated with sedation (Figure 3) [7-9]. This will also allow direct comparison of the two options in those patients as an ancillary study, which would otherwise be unethical. If patients cannot tolerate imaging in the first MR scanner in which they are assigned without claustrophobic reactions, this will be categorized as an event for the primary hypothesis of this project. In case patients cannot undergo imaging in either of the two scanners, conscious sedation according to the standards of the American Society of Anesthesiology will be performed (using Dormicum IV) to enable imaging [17].

Anxiety will be measured using different validated psychological self-report questionnaires such as the Spielberger State-Trait Anxiety Inventory [STAI] [18], the "Fragebogen zu körperbezogenen Ängsten, Kognitionen und Vermeidung [AKV]" [19], the Fear Survey Schedule [FSS-III] [20], and the Claustrophobia Questionnaire [CLQ] [21].

This randomized study has been approved by the local institutional review board at Charité. The clinical experiments will be performed according to the DECLARATION OF HELSINKI - Ethical Principles for Medical Research Involving Human Subjects and the Medizinproduktgesetz (German Medicinal Products Law).

This is an investigator-sponsored industry-independent study.

### Patients

Patients with reported claustrophobia during MR imaging or with inability to undergo MR imaging in conventional scanners because of claustrophobic anxiety will be eligible for the study and subsequent randomization if they have a clinical indication for MR imaging of the head, spine, or shoulder. Patients must not have any absolute or relative contraindication to MR imaging for safety reasons [22], must not have a body weight of more than 200 kg (maximum table weight), will be primarily examined without conscious sedation, and have to be at least 18 years old. Patients must have at least moderate levels of anxiety towards claustrophobic situations as determined using the Claustrophobia Questionnaire [CLQ] [21] (an arithmetic mean of at least 1.0 must be reached by rating anxiety on a scale from 0 to 4 for each of 26 items). All patients will have to give written informed consent before randomization.

### Sample size and statistical analysis

We expect claustrophobia rates in this high-risk patient cohort of 20% in the control group (recent closed short-bore MR imaging) and 5% in the intervention group (open MR imaging). With 82 evaluable patients per group we will achieve the desired power of 80%. Conservatively taking into account an expected drop-out rate of 5%, a total of 174 patients, 87 per group, have to be allocated.

The assumed claustrophobia rates are based on 1) all previous publications on claustrophobia during MR imaging as summarized [3], 2) an expected prevalence of claustrophobic reactions in conventional MR scanners in the study population of at least 60%, and 3) the ability of both open and the recently designed short-bore but closed MR scanners to significantly reduce claustrophobia preventing MR imaging to approximately 5 and 20%, respectively, as indicated in recent nonrandomized pilot studies [3,13,14].

The primary endpoint, claustrophobia that prevents MR imaging, will be evaluated in the intention-to-treat population using a χ^2 ^test. Secondary endpoints will be evaluated by parametric or nonparametric analysis of variance or Mantel-Haenszel tests according to scaling. Appropriate parameters of effect size (e.g., odds ratios) with confidence intervals will be calculated. Subgroups (e.g., sex, age groups, direction of scanning [head-first vs. feet-first]) will be analyzed exploratively.

### Validated questionnaires, MR imaging and need for further improvements

Validated written self-report questionnaires addressing anxiety in general and anxiety related to MR imaging will be used. These include the following: the Spielberger State-Trait Anxiety Inventory [STAI] [18], the "Fragebogen zu körperbezogenen Ängsten, Kognitionen und Vermeidung" [AKV] [19], the Fear Survey Schedule [FSS-III] [20], and the Claustrophobia Questionnaire [CLQ] [21]. Also further nonvalidated custom-designed questionnaires will be used to address patient preferences concerning MR scanner design and which specific scanner design they consider most attractive (Figure 1, Table 1). This information is important to be able to define which further improvements by vendors might be necessary to reduce claustrophobia during MR imaging. Furthermore, patient characteristics, patient satisfaction, clinical outcome, and development of claustrophobia will be addressed (Table 2 and 3). All patients will be asked to again complete some of the questionnaires 4-6 weeks and 7 months after MR imaging. Patients can send back the completed questionnaires to the study coordinator using prepaid envelopes that are sent together with the questionnaires.

**Table 2:** Summary of items addressed in the custom-made questionnaire for initial assessment

Item	Range
School graduation	German secondary schools (Hauptschule, Realschule, Gesamtschule)/Grammar school/GDR schools (EOS, POS)
Leaving school after grade	Free text
Last or current profession	If not employed: Pensioner/Unemployed
Gross family income per month	€0-500/500-1000/1000-1500/1500-2000/2000-2500/2500-3000/3000-4000/4000-5000/> 5000
Marital status	Single/Living with partner/Married/Divorced/Living apart/Single mother or father/Widowed
Number of Children/Number of children under 18 years	Free text
Current extent of claustrophobia	Horizontal visual analogue scale of 10 cm (0-100)
Claustrophobia during MR imaging in the past	Yes/No/If yes description
Claustrophobia preventing prior scheduled MR imaging	Yes/No/If yes description
When did claustrophobic anxiety occur for the first time	Free text
Ideas or strategies to cope with anxiety before and during MR imaging	Yes/No/If yes description*
Belief in successful completion of MR imaging (self-efficacy)	Yes/No/Maybe
Fear of diagnostic findings by MR imaging	Yes/No
Medication	Yes/No/If yes which drugs and dosage
Psychosomatic or psychiatric diseases	Yes/No/If yes which diseases
Previous or current psychotherapy	Yes/No/If yes what kind and how long

*Coping strategies will be categorized according to the Revised Ways of Coping Checklist (RWCCL) [29].

Abbreviations:

EOS = "Erweiterte Oberschule"

GDR = German Democratic Republic

POS = "Polytechnische Oberschule"

**Table 3:** Summary of items addressed in the custom-made follow-up questionnaire

Item	Range
**Examination, Result, Treatment**	
MR findings have revealed the reason for current medical condition (from patients' point of view)	Yes/No
The diagnostic findings obtained by MR imaging have already been known	Yes/No
The diagnostic findings have led to consequences in treatment	Yes/No/If Yes: Conservative treatment/Surgery/Other (patients are asked to add the medical report or the name of the attendant doctor/hospital)
Additional diagnostic findings by other examinations	Yes/No/If yes, which diagnoses (patients are asked to add the medical report or the name of the attendant doctor/hospital)
Improvement of medical condition during the last 7 months	Yes/No/If yes: Without treatment/After treatment/After another measure
**Claustrophobia**	
Current extent of claustrophobia	Horizontal visual analogue scale of 10 cm (0-100)
Attempts to alleviate claustrophobia during the last 7 months	Yes/No/If yes: Psychotherapy/Drugs/Own problem-solving methods
Willingness to start therapy if possible	Yes/No
Time willing to investigate in ambulant or stationary therapy	Nothing/Up to how much time per month/year
Money willing to investigate in therapy (in addition to health insurance contribution)	Nothing/Up to how much money per month/year
**Satisfaction**	
Initial assessment and information about the study	Very good/Good/Satisfactory/Fair/Not at all
Option to see the MR scanners prior to imaging	Very good/Good/Satisfactory/Fair/Not at all
Information about MR imaging	Very good/Good/Satisfactory/Fair/Not at all
MR imaging	Very good/Good/Satisfactory/Fair/Not at all
Delivery of diagnostic findings	Very good/Good/Satisfactory/Fair/Not at all
Further MR imaging	Yes/No/If Yes, of which anatomical region and why
Hypothetical willingness to take part in the study again	Yes/No
Willingness to undergo another MR imaging at the Department of Radiology at Charité Berlin	Yes/No
Would you recommend others to have a scheduled MR imaging at the Department of Radiology at Charité Berlin	Yes/No
Requests, comments, criticism	Free text

### Initial assessment

Immediately after enrollment, patients will complete the above-mentioned questionnaires (German versions of STAI [18], AKV [19], FSS-III [20], and CLQ [21]; and a custom-made questionnaire addressing patient characteristics, Table 2). As depression has been shown to influence anxiety-coping in phobic patients [23], and anxiety can be a symptom of depressive disorders, depression symptoms will be explored using the revised Beck Depression Inventory [BDI-II] [24]. General health-related quality of life will be assessed using the EuroQol questionnaire [EQ-5D] [25]. All questionnaires which will be used for initial assessment are described below.

#### The Claustrophobia Questionnaire [CLQ] [21]

The German version of the Claustrophobia Questionnaire consists of 26 items which assess two separate but related fears hypothesized to comprise claustrophobia: the fear of suffocation (e.g., "having a bad cold and finding it difficult to breathe through your nose") and the fear of restriction (e.g., "locked in a small dark room without windows for 15 min"). For each item anxiety is rated on a scale from 0 (not at all anxious) to 4 (extremely anxious). Summary test scores range from 0 (no anxiety) to 104 (maximum anxiety). The CLQ will be used to determine patients' levels of anxiety toward claustrophobic situations. As an inclusion criterion patients must reach an arithmetic mean of at least 1.0 in the CLQ (equal to a summary score of at least 26) to be eligible for randomization.

#### The Spielberger State-Trait Anxiety Inventory [STAI] [18]

The German version of the Spielberger State-Trait Anxiety Inventory is based on two questionnaires (State and Trait), each with 20 items to be rated on a scale from 1 (almost never) to 4 (very much so). The items are short statements, e.g., "I feel nervous and restless". The State questionnaire consists of 10 positive and 10 negative items which assess the current state of anxiety. The Trait questionnaire consists of 13 positive and 7 negative items assessing the general state of anxiety. Summary test scores range from 20 (no anxiety) to 80 (maximum anxiety).

#### The Fear Survey Schedule [FSS-III] [20]

The Fear Survey Schedule was originally developed to assess change in phobic behavior and generalized anxiety in conditioning therapy. The revised and extended FSS-III has been designed for clinical use. The version of the FSS-III used here consists of 78 items measuring fear, phobic behavior, and generalized anxiety. Each item is a phrase of 2-6 words (e.g., "Being in an elevator") for which a person rates anxiety on a scale from 1 (not at all) to 4 (very much). There are 6 subclassifications of fear in the FSS-III: animal fears (9 items), social and interpersonal fears (18 items), fears of tissue damage, illness, death and their associations (19 items), noise fears (4 items), other classical phobias (20 items), and miscellaneous fears such as strange places, falling, failure, imaginary creatures, strange shapes, feeling angry, dull weather, or making mistakes (8 items). According to Lukins et al. 9 items of the FSS reflect a range of aversive characteristics of the MR imaging procedure: vacuum cleaner noise, being alone, loud noises, being in an elevator, enclosed places, and journeys by airplane [26]. Summary fear scores range from 0 to 312.

#### The „Fragebogen zu körperbezogenen Ängsten, Kognitionen und Vermeidung [AKV]" [19]

The AKV is the German version of the Body Sensations Questionnaire (BSQ), the Agoraphobic Cognitions Questionnaire (ACQ), and the Mobility Inventory (MI) of Chambless et al. [27]. The AKV consists of three systems for the diagnosis of anxiety and somatoform disorders. Each system assesses different symptoms of anxiety and somatoform disorders:

- The "Fragebogen zur Angst vor körperlichen Symptomen" (BSQ, German version) explores the fear of body sensations as internal triggers of anxiety. The questionnaire consists of 17 items (e.g., "heart palpitations") for which anxiety is rated on a scale from 1 (not at all) to 5 (extremely). Summary scores range from 17 to 85. Patients will be asked to report which three of the 17 body sensations occur most frequently when they are anxious or nervous.

- The "Fragebogen zu angstbezogenen Kognitionen" (ACQ, German version) explores the tendency to catastrophizing cognitions and apprehensions which can come along with body sensations. There are two subscales: the "loss of control" subscale (e.g., "I am going to go crazy") and the "physiological consequences" subscale (e.g., "I will have a heart attack"), each with 7 items. For all items occurrence in situations of anxiety is rated on a scale from 1 (never) to 5 (always). Summary scores range from 14 to 70. Patients will be asked to report which three of the 14 cognitions occur most frequently when they are anxious or nervous.

- The "Mobilitätsinventar" (MI, German version) explores the extent and complexity of phobic avoidance behavior referring to external triggers of anxiety. The questionnaire consists of 27 items describing the most important agoraphobic situations (e.g., "elevators"). For each item avoidance is rated twice for "being alone" and "in company" on a scale from 1 (never) to 5 (always). Summary scores range from 54 to 270.

#### The Beck Depression Inventory (BDI-II) [24]

The Beck Depression Inventory is one of the most widely used self-report measures of depression. The German version of the revised Beck Depression Inventory (BDI-II) consists of 21 items comprising the diagnostic criteria for Major Depressive Disorder according to the Diagnostic and Statistical Manual of Mental Disorders [28]. Patients have to rate how they have been feeling for the past two weeks. Each of the 21 items corresponds to a symptom of depression (e.g., concentration difficulty, loss of energy). On 19 items there are 4 statements assessing different severity codes of a symptom (ranging from 0-3). On two items (16 and 18) there are 7 options (though ranging from 0-3) to assess an increase or decrease of appetite and sleep. A summary score of 0-13 is considered minimal depression, 14-19 mild depression, 20-28 moderate depression, and 29-63 severe depression.

#### The EuroQol (EQ-5D) [25]

The EuroQol is a generic non-disease-specific instrument for valuing general health-related quality of life. The questionnaire includes single item measures of five health dimensions: mobility, self-care, daily activities, pain and discomfort, anxiety and depression. Each item has three response options (no problem, some or moderate problems, extreme problems) to rate patients' perceived health status. In addition, the EuroQol includes a vertical visual analogue scale ranging from 0 (worst imaginable) to 100 (best imaginable) to rate the current state of health.

#### Custom-made questionnaire for initial assessment

The custom-made questionnaire for initial assessment addresses patient characteristics such as marital status, gross family income, and claustrophobia during previous MR imaging. The items addressed here are summarized in Table 2.

### MR imaging and image analysis

#### MR imaging

MR imaging will be performed in the supine position using phased-array coils. On the open MR scanner the following coils will be used: a large body coil for imaging of the spine, a small flex coil for imaging of the shoulder, and, to alleviate the feeling of confinement, the neck coil will be used for imaging of the head (in an upside-down position) making use of the elliptical coil design available. If the neck coil is not tolerated for head imaging on the open MR scanner, a multipurpose flex coil can be used as a second alternative. On the closed short-bore MR scanner the spine-array coils that are integrated into the table will be used for imaging of the spine, a small 1-channel circular polarized flex coil will be used for imaging of the shoulder, and a 12-channel bird-cage head coil will be used for imaging of the head. For imaging of the cervical spine, the shoulder, and the head, patients will be examined head-first on both scanners because feet-first imaging, which alleviates claustrophobia by a factor of more than 10 [3], is not possible for these diagnostic questions. For lumbar spine imaging, a feet-first approach can and thus will be utilized on both scanners. Contrast-enhanced sequences will only be obtained if clinically indicated. On the following pages the MR sequences and their characteristics are listed allowing a convenient comparison of the sequence approaches used on both MR scanners for imaging of the head, spine (cervicothoracic, and thoracolumbar), and shoulder (Tables 4, 5, 6, and 7). For detailed information on the sequence characteristics see Additional files 1, 2, 3, and 4. The primary aim of the sequence setup is to obtain a voxel size that is as similar as possible on both scanners. Differences in acquisition time might result from this aim due to different field strengths and gradients.

**Table 4:** Head MR imaging sequences*

	Magnetom Avanto	Panorama
**Basic Sequences**		
Generic sequence name	T1w SE axial	
Vendor sequence name	T1 SE tra	T1w SE
Resulting voxel size (mm)	0.9 × 0.9 × 5.0	0.9 × 0.9 × 5.0
Acquisition time (min:sec)	3:48	4:13
Generic sequence name	PD+T2w TSE axial	
Vendor sequence name	PD+T2 TSE tra	Dual TSE
Resulting voxel size (mm)	0.9 × 0.9 × 5.0	0.9 × 1.0 × 5.0
Acquisition time (min:sec)	3:50	4:15
Generic sequence name	TIRM axial	
Vendor sequence name	T2 TIRM tra dark-fl	T2w FLAIR
Resulting voxel size (mm)	0.9 × 0.9 × 5.0	0.9 × 1.0 × 5.0
Acquisition time (min:sec)	3:38	6:36
Optional Sequences**		
Generic sequence name	DWI axial	
Vendor sequence name	Ep2 D diff3 scan trace	DWI
Resulting voxel size (mm)	1.8 × 1.8 × 5.0	1.9 × 1.9 × 5.0
Acquisition time (min:sec)	0:48	0:58
Generic sequence name	T2w Star axial	
Vendor sequence name	T2 fl2 D tra hemo	T2w FFE
Resulting voxel size (mm)	0.9 × 0.9 × 5.0	0.9 × 0.9 × 5.0
Acquisition time (min:sec)	3:01	5:08
Generic sequence name	T1w MPRAGE post contrast agent	
Vendor sequence name	T1 fl3Dsag	T1w 3 D FFE
Resulting voxel size (mm)	0.9 × 0.9 × 1.0	1.0 × 1.2 × 1.0
Acquisition time (min:sec)	6:49	7:44

*For further information on the sequence characteristics see Additional file 1.

**These sequences will only be acquired if a clinical indication (e.g., for contrast-enhanced T1-weighted sequences) exists. In all patients, however, the basic sequences listed above will be obtained.

Abbreviations:

Dual = Double Echo

DWI = Diffusion-Weighted Imaging

EPI = Echo Planar Imaging

FFE = Fast Field Echo

FLAIR = FLuid Attenuation Inversion Recovery

MPRAGE = Magnetization Prepared RApid Gradient Echo

PD = Proton Density

Sag = sagittal

SE = Spin Echo

T1w = T1-weighted

T2w = T2-weighted

TIRM = Turbo Inversion Recovery Measurement

Tra = transverse

TSE = Turbo Spin Echo

**Table 5:** Cervicothoracic spine MR imaging sequences*

	Magnetom Avanto	Panorama
**Basic Sequences**		
Generic sequence name	T2w sagittal	
Vendor sequence name	T2 TSE rst sag	T2w TSE
Resulting voxel size (mm)	0.9 × 1.0 × 3.0	1.1 × 1.1 × 3.0
Acquisition time (min:sec)	3:25	5:09
Generic sequence name	T1w sagittal	
Vendor sequence name	T1 TSE sag	T1w TSE
Resulting voxel size (mm)	1.0 × 1.5 × 3.0	1.1 × 1.5 × 3.0
Acquisition time (min:sec)	4:01	4:15
Generic sequence name	T2w axial	
Vendor sequence name	T2 me2 D tra	3 D mFFE
Resulting voxel size (mm)	1.0 × 1.0 × 3.0	1.0 × 1.0 × 3.0
Acquisition time (min:sec)	6:53	7:38
Optional Sequences**		
Generic sequence name	TIRM	
Vendor sequence name	TIRM	STIR TSE
Resulting voxel size (mm)	1.2 × 1.5 × 3.0	1.2 × 1.7 × 3.0
Acquisition time (min:sec)	5:42	5:56
Generic sequence name	T1w sagittal post contrast agent	
Vendor sequence name	T1 TSE sag	T1w TSE
Resulting voxel size (mm)	1.5 × 1.0 × 3.0	1.1 × 1.5 × 3.0
Acquisition time (min:sec)	4:01	4:15
Generic sequence name	T1w axial post contrast agent	
Vendor sequence name	T1 TSE tra	T1w TSE
Resulting voxel size (mm)	1.0 × 1.2 × 3.0	1.0 × 1.2 × 3.0
Acquisition time (min:sec)	2:16	4:12

*For further information on the sequence characteristics see Additional file 2.

**These sequences will only be acquired if a clinical indication (e.g., for contrast-enhanced T1-weighted sequences) exists. In all patients, however, the basic sequences listed above will be obtained.

Abbreviations:

FFE = Fast Field Echo

Me = medic

Sag = sagittal

STIR = Short T1 Inversion Recovery

T1w = T1-weighted

T2w = T2-weighted

TIRM = Turbo Inversion Recovery Magnitude

Tra = transverse

TSE = Turbo Spin Echo

**Table 6:** Thoracolumbar spine MR imaging sequences*

	Magnetom Avanto	Panorama
**Basic Sequences**		
Generic sequence name	T2w sagittal	
Vendor sequence name	T2 TSE rst sag	T2w TSE
Resulting voxel size (mm)	0.9 × 1.0 × 3.0	1.1 × 1.1 × 3.0
Acquisition time (min:sec)	3:25	5:09
Generic sequence name	T1w sagittal	
Vendor sequence name	T1 TSE sag	T1w TSE
Resulting voxel size (mm)	1.0 × 1.5 × 3.0	1.1 × 1.5 × 3.0
Acquisition time (min:sec)	4:01	4:15
Generic sequence name	T2w axial	
Vendor sequence name	T2 TSE rst tra	T2w TSE
Resulting voxel size (mm)	0.6 × 0.8 × 4.0	0.7 × 0.8 × 4.0
Acquisition time (min:sec)	2:23	2:45
Optional Sequences**		
Generic sequence name	TIRM sagittal	
Vendor sequence name	TIRM sag	STIR TSE
Resulting voxel size (mm)	1.2 × 1.5 × 3.0	1.2 × 1.7 × 3.0
Acquisition time (min:sec)	5:42	5:56
Generic sequence name	T1w sagittal post contrast agent	
Vendor sequence name	T1 TSE sag	T1w TSE
Resulting voxel size (mm)	1.0 × 1.5 × 3.0	1.1 × 1.5 × 3.0
Acquisition time (min:sec)	4:01	4:15
Generic sequence name	T1w axial post contrast agent	
Vendor sequence name	T1 TSE tra	T1w TSE
Resulting voxel size (mm)	1.0 × 1.2 × 4.0	1.0 × 1.2 × 4.0
Acquisition time (min:sec)	3:22	3:30

*For further information on the sequence characteristics see Additional file 3.

**These sequences will only be acquired if a clinical indication (e.g., for contrast-enhanced T1-weighted sequences) exists. In all patients, however, the basic sequences listed above will be obtained.

Abbreviations:

Sag = sagittal

STIR = Short T1 Inversion Recovery

T1w = T1-weighted

T2w = T2-weighted

TIRM = Turbo Inversion Recovery Magnitude

Tra = transverse

TSE = Turbo Spin Echo

**Table 7:** Shoulder MR imaging sequences*

	Magnetom Avanto	Panorama
**Basic Sequences**		
Generic sequence name	T1w axial	
Vendor sequence name	T1 tra	T1w TSE
Resulting voxel size (mm)	0.9 × 0.7 × 3.5	0.7 × 0.9 × 3.5
Acquisition time (min:sec)	2:17	2:47
Generic sequence name	T2w axial	
Vendor sequence name	T2 me2 D tra	3 D mFFE WATS
Resulting voxel size (mm)	0.7 × 1.0 × 3.5	0.7 × 1.0 × 3.5
Acquisition time (min:sec)	3:19	7:11
Generic sequence name	TIRM coronal	
Vendor sequence name	T1 TIRM cor	STIR
Resulting voxel size (mm)	0.6 × 0.8 × 4.0	0.6 × 0.9 × 4.0
Acquisition time (min:sec)	5:15	6:36
Generic sequence name	PD+T2w TSE coronal	
Vendor sequence name	PD+T2 TSE cor	Dual dr tsetse
Resulting voxel size (mm)	0.6 × 0.8 × 4.0	0.6 × 0.8 × 4.0
Acquisition time (min:sec)	4:16	5:59
Generic sequence name	T2w TSE parasagittal	
Vendor sequence name	T2 TSE rst fs parasag	T2 TSE SPIR
Resulting voxel size (mm)	0.7 × 0.9 × 4.0	0.7 × 0.9 × 4.0
Acquisition time (min:sec)	4:04	4:52

*For further information on the sequence characteristics see Additional file 4.

Abbreviations:

Cor = coronal

Dual = Double echo

FFE = Fast Field Echo

Me = medic

Parasag = parasagittal

PD = Proton Density

Sag = sagittal

SPIR = Spectral Presaturation Inversion Recovery

STIR = Short T1 Inversion Recovery

T1w = T1-weighted

T2w = T2-weighted

TIRM = Turbo Inversion Recovery Magnitude

Tra = transverse

TSE = Turbo Spin Echo

WATS = WATer only Selection

To assess patients' anxiety before and during MR imaging the State questionnaire of the State-Trait Anxiety Inventory will be used directly before and after MR imaging. The questionnaire which will be used after imaging is reworded in the past tense to assess patients' anxiety during the MR scan. This questionnaire will not be administered if the MR procedure is terminated before starting the scan (Figure 3).

Patients' strategies to cope with their anxiety will be assessed according to the Revised Ways of Coping Checklist (RWCCL) [29]. The RWCCL is a self-report measure of coping which is based on Lazarus' transactional model of stress [30]. The questionnaire consists of five scales: problem-focused coping (15 items, e.g., "made a plan of action and followed it"), wishful thinking (8 items, e.g., "daydreamed or imagined a better time or place than the one I was in"), seeks social support (6 items, e.g.,"talked to others and accepted their sympathy"), avoidance (10 items, e.g., "tried to make myself feel better by eating, drinking, smoking, taking medications") and blamed self (3 items, e.g., "blamed yourself). The RWCCL will not be used as a self-report measure, but instead coping strategies will be assessed by a study assistant, who will not influence interactions between patients and radiology technicians (radiographers). Items not conforming to MR imaging will be excluded from this questionnaire.

Furthermore, a study assistant will record important information in an MR imaging questionnaire: scan duration, claustrophobic events, the anatomical region examined, the use of optional sequences and/or contrast agent, the names of all staff members interacting with the patient, assistance by the staff, any complications or interruption during the scan, the kind of ear protection (headphones, earplugs or none on the noise-reduced MR scanner) as well as sedation information.

#### MR image analysis

MR images will be analyzed by two blinded readers on a workstation (Centricity PACS Workstation RA 1000, GE Healthcare). Details of the MR image analysis can be found in the image analysis forms (Additional files 5, 6, and 7).

Qualitative image analysis will be performed via grading from 1 to 5. The scale will be 1 = optimal, 2 = good, 3 = moderate, 4 = poor, 5 = nondiagnostic to rate contrast, contour sharpness, and overall image quality and 1 = none, 2 = minimal, 3 = moderate, 4 = major, 5 = nondiagnostic to rate artifacts and noise. Artifacts will be classified as being due to motion, pulsation, metal, noise, or other. In shoulder imaging the fatty infiltration grade of cuff muscles will be determined on a scale from 0 to 4 (0 = no fat, 1 = a few streaks of fat, 2 = more muscle than fat, 3 = as much muscle as fat, 4 = more fat than muscle) according to Goutallier et al. [31], and the grade of muscle atrophy of cuff muscles will be determined on a scale from 0 to 4 (0 = none, 2 = mild, 3 = moderate, 4 = severe) according to Warner et al. [32]. In addition, the permitted diagnostic quality of assessment of rotator cuff muscles and their ligaments (coronal imaging) and the glenoid labrum (axial imaging) will be determined via grading from 0 to 5 (1 = optimal, 2 = good, 3 = moderate, 4 = poor and 5 = nondiagnostic).

Quantitative image analysis will be performed via measurements of signal intensities (SI) in circular regions of interest (ROI) ranging in size from 0.02 up to 0.4 cm² standardized according to the anatomy. Mean values and standard deviations will be calculated for each measurement in each standard sequence of either head, spine, or shoulder MR imaging. Standard deviations (SD) of the ROI signals will be used to measure noise, and signal-to-noise and contrast-to-noise ratios were calculated as recently described [33]. Furthermore, contour clarity will be analyzed as recently described [34], using ImageJ http://rsbweb.nih.gov/ij/. Contour clarity in head imaging will be distinguished between corticospinal fluid, caudate nucleus, and genu of corpus callosum. In spine imaging, contour clarity will be determined for gray and white matter of the spinal cord, corticospinal fluid, and vertebral body. In shoulder imaging, it will be determined for joint fluid and glenoid labrum (axial images), for fat and muscle (deltoideus), and for bone marrow (humerus head).

### Need for further improvements

A custom-made questionnaire will be used to track certain features of the MR procedure (e.g., pain and noise) and patient preferences concerning MR scanner design and which specific scanner design patients consider most attractive (Figure 1). This information is important to be able to define which further improvements by vendors might be necessary to reduce claustrophobic reactions during MR imaging. In Table 1 we have summarized the main objectives addressed in this questionnaire.

### Follow-up after 4 to 6 weeks

At the initial follow-up, the STAI [18], AKV [19], FSS-III [20], and CLQ [21] questionnaires will be repeated. The State questionnaire of the STAI will be repeated in two versions assessing the current state of anxiety and the state of anxiety during MR imaging (if accomplished without sedation).

### Follow-up after 7 months

At the final follow-up, the STAI [18], AKV [19], FSS-III [20], CLQ [21], BDI-II [24], and EuroQol [25] questionnaires will be repeated. The State questionnaire of the STAI will be repeated in two versions assessing the current state of anxiety and the state of anxiety during MR imaging (if accomplished without sedation). Moreover, a custom-made questionnaire will be administered to assess clinical outcome, development of claustrophobic anxiety, and general satisfaction with the study participation. The items addressed here are summarized in Table 3.

## Discussion

Patients with claustrophobic anxiety, who are more likely to be frightened during MR imaging, have been reported to experience claustrophobia in up to 35% of all examinations [6]. To complete the scan, conscious sedation and additional sequences (after sedation) may be necessary in these patients. This situation involves significant risks of adverse events [7-9] and is associated with additional costs because it reduces workflow, limits patient acceptance, and wastes valuable scanning time and thus induces costs. On the other hand, prematurely terminated scans as well mean an important financial loss for the health care system. Improved patient comfort during MR imaging (mainly reduction of noise and sensation of confinement) appears to be essential to avoid claustrophobic reactions [10,11].

A more patient-centered design of closed MR systems is one effective approach to make MR imaging more comfortable for patients. Few studies have investigated patient acceptance and the potential to reduce claustrophobic anxiety of recent closed MR scanners with noise reduction and short- and wide-bore design in comparison to conventional MR scanners.

In a large cohort study of 55,734 outpatients, Dewey et al. assessed the potential to reduce claustrophobia of a recent 1.5-T MR scanner with 97% noise reduction and a short- and wide-bore design [3]. Patients were examined on either the recent or a conventional MR scanner. The recent MR scanner was shown to reduce claustrophobia by a factor of 3, although there were more head-first examinations on that scanner. Intraindividual comparisons of patients who underwent imaging on both MR units showed similar results with a claustrophobia rate of below 1% [3].

In a retrospective study on the occurrence of claustrophobia in 5,682 patients examined on either a 1.5-T closed MR scanner or a 0.5-T noise-reduced MR scanner with a short and wide bore Dantendorfer et al. found no significant difference between the two MR units [35]. However, there was a selection bias because staff was referring highly anxious patients to the recent MR scanner. Thus, the authors conclude that the short-bore MR scanner may in fact reduce claustrophobia [35]. In a later study by Dantendorfer et al., 297 patients without MR imaging experience were randomly examined on either a 1.5-T closed MR scanner or a recent 0.5-T noise-reduced MR scanner with a short and wide bore [36]. Anxiety before and after the scan was among others assessed using the State questionnaire of the STAI. Although patients felt more at ease in the recent MR scanner (concerning noise and confinement), there was no significant difference in patient acceptance and occurrence of motion artifacts. Interestingly, there was no correlation between STAI scores and motion artifacts, but patients who were more concerned about the technical apparatus had more motion artifacts [36].

Another approach to improve patient acceptance of MR imaging is a more open vertical scanner configuration, although until recently such systems operated at rather low field strengths (0.2 T), resulting in poor image quality.

Heuck et al. compared two high-field closed MR systems operating at 1.5-T and 1.0-T with an open whole-body scanner and a dedicated extremity MR system, both operating at 0.2-T [37]. In 40 patients examined on each system (160 total) they found a mainly positive condition and acceptance for all MR scanners. However, patients who were examined on the open MR scanners felt significantly more confident and calm, despite longer examination durations. Patients' suggestions for improvement of the high-field closed MR systems mainly concerned reduction of noise and enlargement of the bore. Nevertheless, these authors concluded that the necessary technical conditions and not differences in patient preference should be crucial for the choice of an MR system [37].

In a recent pilot study Bangard et al. have examined 36 claustrophobic and 36 non-claustrophobic patients on a recently introduced open MR scanner with high-field strength (1.0 T) [14]. Anxiety was assessed using validated questionnaires (STAI, CLQ, MRI FSS [26]). In the claustrophobic patients scan termination rate was reduced to 8% compared to 58.3% in previous examinations on conventional MR systems, and 91.7% preferred the open MR scanner. Furthermore, claustrophobia did not reduce image quality by motion artifacts [14].

A 0.5-T interventional MR scanner with a vertical gap in the bore of the magnet ("double donut") was used in a study by Spouse et al. [13]. 96% of 50 claustrophobic patients who were unable to complete a conventional MR scan successfully underwent MR imaging on the interventional scanner. Patients reported less anxiety than during their previous MR imaging and felt significantly better informed about the procedure. However, friends or relatives were allowed to stay in the magnet room and many patients indicated that this, beside the scanner design, had helped them considerably [13].

In contrast to the above mentioned studies, the CLAUSTRO trial will directly compare an open MR scanner with a vertical 1.0-T magnetic field, and a closed noise-reduced MR scanner with a short- and wide-bore design and 1.5-T field strength in regards to claustrophobia and image quality as well as influence on further clinical management in a randomized controlled trial. Patients included will have at least moderate levels of claustrophobic anxiety, experienced claustrophobia during a prior MR imaging or rejected a current scheduled MR imaging due to claustrophobia, and will have a clinical indication for MR imaging and will thus be at high risk to experience claustrophobia during the MR scan. Anxiety will be assessed before and after MR imaging as well as 4-6 weeks and 7 months after the scan in order to address the development of claustrophobia. Increase of claustrophobic anxiety has been found if the scan is terminated prematurely or high anxiety is tolerated [26,38-40]. On the other hand, there are reports of decreased anxiety after completed MR imaging [40,41]. Long-term alleviation of claustrophobia after completed MR imaging might support the potential of exposure therapy to treat claustrophobia [42,43]. To perform the comparison of image quality as reliable as possible the anatomical regions to be examined will be limited to head, spine and shoulder, and equal sequences on both scanners with comparable voxel sizes will be utilized. Diagnostic utility and clinical outcome will be assessed 7 months after MR imaging which is an appropriate time-frame to detect an influence on clinical management. Of course this will be mediated through the patients and referring physicians' response to MR results. Furthermore, the study will address which specific scanner design is evaluated by patients to be the most attractive and which further improvements in designing MR scanners should be followed by manufacturers. Randomization will be done as the most decisive evidence for judging the efficacy of diagnostic tests comes from randomized comparisons in which the controlled design obviates the biases of observational studies [15,16]. Patients who are assigned to imaging in one MR scanner but cannot bear this procedure due to claustrophobic anxiety will be cross-referred to imaging in the other scanner in order to preclude adverse events associated with sedation [7-9]. This will also allow direct comparison of the two options in those patients as an ancillary study. Due to its design this trial will also be able to analyze and compare the cost-effectiveness of the two MR scanners.

### Study limitations

Anxiety during MR imaging can occur due to several other psychosomatic or psychiatric disorders than claustrophobia e.g., panic disorder, adjustment disorder with anxious mood, or major depression [10]. Within the limits of the study, claustrophobia will not be diagnosed according to ICD-10 or DSM-IV criteria [28,44]. Furthermore, there are several features of MR imaging and the patient which can contribute to anxiety beyond confinement by the MR scanner. Such features may be pain, loud noise, the unknown, concerns about diagnostic findings, having to lie still, the examination duration, uncomfortable temperature or the MR scanner itself [5,11,45-47]. Several studies have shown that especially pain is correlated with anxiety during the scan and premature termination [11,38,40]. However, claustrophobia will be assessed using the CLQ [21], and reaching a specific score is an inclusion criterion (an arithmetic mean of greater or equal 1.0 must be reached by rating anxiety on a scale from 1 to 4 for each of 26 items). Furthermore, a custom-made questionnaire addressing prior claustrophobic events before or during scheduled MR imaging and first occurrence of claustrophobia will be fulfilled by a study assistant in the manner of a structured interview. Further questionnaires will help to examine generalized anxiety, patients' health status (including pain) and depression symptoms. The pain and noise levels which patients experience during MR imaging will be assessed directly after the scan by using horizontal visual analogue scales and patients will be asked to report whether they are afraid of diagnostic findings in MR imaging. Claustrophobia as part of e.g., a panic disorder is no exclusion criterion. However, patients will be asked to report whether they have known psychosomatic or psychiatric disorders. In general, in the majority of articles addressing anxiety leading to premature termination of MR imaging, anxiety is referred to as "claustrophobia" [5]. For the assessment of anxiety during MR imaging it is also important to know whether patients take any anxiolytic or sedative drugs or other psychotropics which will thus be examined. Different examination times (due to different sequence duration, anxiety or complications) and different coils will also be assessed.

Another feature of MR imaging whose influence on patients' condition will be difficult to measure is the support by nursing stuff, radiographers and physicians. Several studies have shown the importance of social support and the appreciation of a caring attitude of others by anxious patients [13,47-49]. It might yet influence patients to know that staff is aware of their anxiety and the staff may act more caring because of this awareness. However, patients' seeking social support will be assessed by evaluating coping strategies and patients will be asked to report what did particularly help them to cope with their anxiety. Furthermore, the staff will be instructed not to be "overprotective" and not to suggest coping strategies to the patients. Of course, patients will experience more attendance than in clinical routine. To keep the influence as constant as possible, patients will mainly be attended by two female staff members throughout the study and there will be a limited number of physicians and radiographers who perform MR imaging.

With regard to the comparison of image quality, the primary aim of the sequence set-up is to obtain a voxel size that is as similar as possible on both MR scanners. Thus, compromises have to be made concerning the best possible image quality still providing comparability. Motion artifacts due to anxiety might also play a role [45,50] but should be obviated by the high number of patients and randomization.

Concerning the closed MR scanner with short- and wide-bore design which will be used in this study, it should be mentioned that there are scanners with even shorter and wider bores like the Siemens Magnetom Espree with 0,7 m diameter and 1,25 m length of the scanner bore. However, this improvement in scanner design affects the image quality which can be achieved with this MR scanner.

## Conclusions

This randomized controlled trial will be the first direct comparison of open vertical and closed short-bore MR systems in regards to claustrophobia and image quality as well as diagnostic utility. If an open MR scanner can be shown to reduce claustrophobia in a randomized comparison it might be justified to recommend this approach for routine clinical application in certain high-risk anxiety patients or in certain imaging centers. Furthermore, it is the first study to address which specific scanner design is evaluated by patients to be the most attractive for imaging. This knowledge is important to be able to define which further improvements by the vendors might be necessary to reduce claustrophobic reactions. Accordingly, this randomized trial has the potential to influence both the clinical and economical utilization and impact of MR imaging.

## Competing interests

Dr. Dewey reported receiving grant support from GE Healthcare, Bracco, Guerbet, the European Funds for Regional Development (EFRE), the German Heart Foundation/German Foundation of Heart Research, and Toshiba Medical Systems and lecture fees from Toshiba Medical Systems, Cardiac MR Academy Berlin, Guerbet, and Bayer-Schering; he is also a consultant for Guerbet. Dr. Rief reported receiving travel reimbursement from CMC contrast. Dr. Teichgräber reported grant support from the Technology Foundation Berlin, Philips Medical Systems, and EFRE. Prof. Hamm reported receiving grant support from GE Healthcare, Schering, Siemens Medical Solutions, and Toshiba Medical Systems, and lecture fees from Siemens Medical Solutions and Bayer-Schering. Furthermore, there are institutional master research agreements with Philips Medical Systems, Siemens Medical Solutions, and Toshiba Medical Systems. These funding sources had no role in the collection, analysis, or interpretation of the data or in the decision to submit the manuscript for publication. The other authors reported no financial disclosures.

## Authors' contributions

JE, EZ, and MD drafted the manuscript and participated in the design of the study and its coordination. MR participated in the design of the study and its coordination and helped to draft the manuscript. PM provided statistical expertise and participated in the design of the study. RK, PA, CK, GD, TB, UT, and BH gave administrative, technical, or logistic support and critically revised the article for important intellectual content. MD conceived the study. All authors read and approved the final manuscript.

## Pre-publication history

The pre-publication history for this paper can be accessed here:

http://www.biomedcentral.com/1471-2342/11/4/prepub

## Supplementary Material

Additional file 1 **Appendix Table S1**. Further information on head MR imaging sequences used. Click here for file

Additional file 2 **Appendix Table S2**. Further information on cervicothoracic spine MR imaging sequences used. Click here for file

Additional file 3 **Appendix Table S3**. Further information on thoracolumbar spine MR imaging sequences used. Click here for file

Additional file 4 **Appendix Table S4**. Further information on shoulder MR imaging sequences used. Click here for file

Additional file 5 **Appendix Figure S1**. MR image analysis form for quantitative and qualitative analysis of head imaging. Click here for file

Additional file 6 **Appendix Figure S2**. MR image analysis form for quantitative and qualitative analysis of spine imaging. Click here for file

Additional file 7 **Appendix Figure S3**. MR imaging analysis form for quantitative and qualitative analysis of shoulder imaging. Click here for file
