# Supplementary material for: Reduction of claustrophobia during magnetic resonance imaging: methods and design of the "CLAUSTRO" randomized controlled trial
Source: BMC Med Imaging. 2011 Feb 10;11:4. doi: 10.1186/1471-2342-11-4 (PMC3045881; doi:10.1186/1471-2342-11-4)
Supplement: Additional file 1 — Appendix Table S1. Further information on head MR imaging sequences used. [file 1471-2342-11-4-S1.PDF]

Appendix Table 1. Head MR imaging Sequences Used

		Magnetom Avanto		Panorama
**Basic Sequences**				
Generic sequence name			T1w SE axial	
	Vendor sequence name	T1 SE tra		T1w SE
	TR (ms)	500		575
	TE (ms)	7.8		15
	Slices	21		21
	Slice thickness (mm)	5.0		5.0
	Resulting voxel size (mm)	0.9 x 0.9 x 5.0		0.9 x 0.9 x 5.0
	Averages	2		2
	Acquisition time (min:sec)	3:48		4:13
Generic sequence name			PD+T2w TSE axial	
	Vendor sequence name	PD+T2 TSE tra		Dual TSE
	TR (ms)	4660		2200
	First TE (ms)	12		11
	Second TE (ms)	119		120
	Slices	21		21
	Slice thickness (mm)	5.0		5.0
	Resulting voxel size (mm)	0.9 x 0.9 x 5.0		0.9 x 1.0 x 5.0
	Averages	2		2
	Turbo factor	9		14
	Acquisition time (min:sec)	3:50		4:15
Generic sequence name			TIRM axial	
	Vendor sequence name	T2 TIRM tra dark-fl		T2w FLAIR
	TR (ms)	9000		6000
	TE (ms)	109		90
	TI (ms)	2500		2000
	Slices	21		21
	Slice thickness (mm)	5.0		5.0
	Resulting voxel size (mm)	0.9 x 0.9 x 5.0		0.9 x 1.0 x 5.0
	Averages	1		2
	Turbo factor	21		21
	Acquisition time (min:sec)	3:38		6:36
**Optional Sequences***				
Generic sequence name			DWI axial	
	Vendor sequence name	Ep2D diff 3scan trace		DWI
	TR (ms)	3100		3878
	TE (ms)	87		98
	Slices	21		21
	Slice thickness (mm)	5.0		5.0
	Resulting voxel size (mm)	1.8 x 1.8 x 5.0		1.9 x 1.9 x 5.0
	Averages	2		3
	EPI factor	112		107
	Acquisition time (min:sec)	0:48		0:58
Generic sequence name			T2w Star axial	
	Vendor sequence name	T2 fl2D tra hemo		T2w FFE
	TR (ms)	800		700
	TE (ms)	26		21
	Slices	21		21
	Slice thickness (mm)	5.0		5.0
	Resulting voxel size (mm)	0.9 x 0.9 x 5.0		0.9 x 0.9 x 5.0
	Averages	1		2
	Acquisition time (min:sec)	3:01		5:08
Generic sequence name			T1w MPRAGE post contrast agent	
	Vendor sequence name	T1 fl3D sag		T1w 3D FFE
	TR (ms)	8.3		12
	TE (ms)	4.76		6.9
	3D stacks; Slices	1; 192		1; 192
	Slice thickness (mm)	1.0		1.0
	Resulting voxel size (mm)	0.9 x 0.9 x 1.0		1.0 x 1.2 x 1.0
	Averages	1		1
	Acquisition time (min:sec)	6:49		7:44

*These sequences will only be acquired if a clinical indication (e.g., for contrast-enhanced T1-weighted sequences) exists. In all patients, however, the basic sequences listed above will be obtained.

Abbreviations:

Dual = Double Echo
DWI = Diffusion-Weighted Imaging
EPI = Echo Planar Imaging
FFE = Fast Field Echo
FLAIR = FLuid Attenuation Inversion Recovery
MPRAGE = Magnetization Prepared RApid Gradient Echo
PD = Proton Density
Sag = sagittal
SE = Spin Echo
T1w = T1-weighted
T2w = T2-weighted
TE = Echo Time
TI = Inversion Time
TIRM = Turbo Inversion Recovery Magnitude
TR = Relaxation Time
Tra = transverse
TSE = Turbo Spin Echo
