# Supplementary material for: Reduction of claustrophobia during magnetic resonance imaging: methods and design of the "CLAUSTRO" randomized controlled trial
Source: BMC Med Imaging. 2011 Feb 10;11:4. doi: 10.1186/1471-2342-11-4 (PMC3045881; doi:10.1186/1471-2342-11-4)
Supplement: Additional file 2 — Appendix Table S2. Further information on cervicothoracic spine MR imaging sequences used. [file 1471-2342-11-4-S2.PDF]

Appendix Table 2. Cervicothoracic spine MR imaging sequences

	Magnetom Avanto	Panorama
**Basic Sequences**		
Generic sequence name	T2w sagittal	
Vendor sequence name	T2 TSE rst sag	T2w TSE
TR (ms)	3840	3819
TE (ms)	109	120
Slices	15	15
Slice thickness (mm)	3.0	3.0
Resulting voxel size (mm)	0.9 x 1.0 x 3.0	1.1 x 1.1 x 3.0
Averages	2	4
Turbo factor	17	20
Acquisition time (min:sec)	3:25	5:09
Generic sequence name	T1w sagittal	
Vendor sequence name	T1 TSE sag	T1w TSE
TR (ms)	610	618
TE (ms)	9.5	11
Slices	15	15
Slice thickness (mm)	3.0	3.0
Resulting voxel size (mm)	1.0 x 1.5 x 3.0	1.1 x 1.5 x 3.0
Averages	4	4
Turbo factor	3	6
Acquisition time (min:sec)	4:01	4:15
Generic sequence name	T2w axial	
Vendor sequence name	T2 me2D tra	3D mFFE
TR (ms)	1430	89
TE (ms)	20	13,8
Slices	34	34
Slice thickness (mm)	3.0	3.0
Resulting voxel size (mm)	1.0 x 1.0 x 3.0	1.0 x 1.0 x 3.0
Averages	2	1
Acquisition time (min:sec)	6:53	7:38
**Optional Sequences***		
Vendor sequence name	TIRM	
Brand sequence name	TIRM	STIR TSE
TR (ms)	3840	2000
TE (ms)	52	50
TI (ms)	160	135
Slices	15	15
Slice thickness (mm)	3.0	3.0
Resulting voxel size (mm)	1.2 x 1.5 x 3.0	1.2 x 1.7 x 3.0
Averages	1	2
Turbo factor	7	6
Acquisition time (min:sec)	5:42	5:56
Generic sequence name	T1w sagittal post contrast agent	
Vendor sequence name	T1 TSE sag	T1w TSE
TR (ms)	610	618
TE (ms)	9.5	11
Slices	15	15
Slice thickness (mm)	3.0	3.0
Resulting voxel size (mm)	1.5 x 1.0 x 3.0	1.1 x 1.5 x 3.0
Averages	4	4
Turbo factor	3	6
Acquisition time (min:sec)	4:01	4:15
Generic sequence name	T1w axial post contrast agent	
Vendor sequence name	T1 TSE tra	T1w TSE
TR (ms)	758	595
TE (ms)	10	10
Slices	34	34
Slice thickness (mm)	3.0	3.0
Resulting voxel size (mm)	1.0 x 1.2 x 3.0	1.0 x 1.2 x 3.0
Averages	2	4
Turbo factor	3	5
Acquisition time (min:sec)	2:16	4:12

*These sequences will only be acquired if a clinical indication (e.g., for contrast-enhanced T1-weighted sequences) exists. In all patients, however, the basic sequences listed above will be obtained.

Abbreviations:

FFE	= Fast Field Echo
Me	= medic
Sag	= sagittal
STIR	= Short T1 Inversion Recovery
T1w	= T1-weighted
T2w	= T2-weighted
TE	= Echo Time
TI	= Inversion Time
TIRM	= Turbo Inversion Recovery Magnitude
TR	= Relaxation Time
Tra	= transverse
TSE	= Turbo Spin Echo
