# Supplementary material for: Reduction of claustrophobia during magnetic resonance imaging: methods and design of the "CLAUSTRO" randomized controlled trial
Source: BMC Med Imaging. 2011 Feb 10;11:4. doi: 10.1186/1471-2342-11-4 (PMC3045881; doi:10.1186/1471-2342-11-4)
Supplement: Additional file 3 — Appendix Table S3. Further information on thoracolumbar spine MR imaging sequences used. [file 1471-2342-11-4-S3.PDF]

Appendix Table 3. Thoracolumbar spine MR imaging sequences

	Magnetom Avanto		Panorama
**Basic Sequences**			
Generic sequence name		T2w sagittal	
Vendor sequence name	T2 TSE rst sag		T2w TSE
TR (ms)	3840		3819
TE (ms)	109		120
Slices	15		15
Slice thickness (mm)	3.0		3.0
Resulting voxel size (mm)	0.9 x 1.0 x 3.0		1.1 x 1.1 x 3.0
Averages	2		4
Turbo factor	17		20
Acquisition time (min:sec)	3:25		5:09
Generic sequence name		T1w sagittal	
Vendor sequence name	T1 TSE sag		T1w TSE
TR (ms)	610		618
TE (ms)	9.5		11
Slices	15		15
Slice thickness (mm)	3.0		3.0
Resulting voxel size (mm)	1.0 x 1.5 x 3.0		1.1 x 1.5 x 3.0
Averages	4		4
Turbo factor	3		6
Acquisition time (min:sec)	4:01		4:15
Generic sequence name		T2w axial	
Vendor sequence name	T2 TSE rst tra		T2w TSE
TR (ms)	6450		3172
TE (ms)	92		120
Slices	24		24
Slice thickness (mm)	4.0		4.0
Resulting voxel size (mm)	0.6 x 0.8 x 4.0		0.7 x 0.8 x 4.0
Averages	1		3
Turbo factor	20		24
Acquisition time (min:sec)	2:23		2:45
**Optional Sequences***			
Generic sequence name		TIRM sagittal	
Vendor sequence name	TIRM sag		STIR TSE
TR (ms)	3840		2000
TE (ms)	52		50
TI (ms)	160		135
Slices	15		15
Slice thickness (mm)	3.0		3.0
Resulting voxel size (mm)	1.2 x 1.5 x 3.0		1.2 x 1.7 x 3.0
Averages	1		2
Turbo factor	7		6
Acquisition time (min:sec)	5:42		5:56
Generic sequence name		T1w sagittal post contrast agent	
Vendor sequence name	T1 TSE sag		T1w TSE
TR (ms)	610		618
TE (ms)	9.5		11
Slices	15		15
Slice thickness (mm)	3.0		3.0
Resulting voxel size (mm)	1.0 x 1.5 x 3.0		1.1 x 1.5 x 3.0
Averages	4		4
Turbo factor	3		6
Acquisition time (min:sec)	4:01		4:15
Generic sequence name		T1w axial post contrast agent	
Vendor sequence name	T1 TSE tra		T1w TSE
TR (ms)	535		565
TE (ms)	10		10
Slices	24		24
Slice thickness (mm)	4.0		4.0
Resulting voxel size (mm)	1.0 x 1.2 x 4.0		1.0 x 1.2 x 4.0
Averages	2		4
Turbo factor	3		3
Acquisition time (min:sec)	3:22		3:30

*These sequences will only be acquired if a clinical indication (e.g., for contrast-enhanced T1-weighted sequences) exists. In all patients, however, the basic sequences listed above will be obtained.

Abbreviations:

Sag	= sagittal
STIR	= Short T1 Inversion Recovery
T1w	= T1-weighted
T2w	= T2-weighted
TE	= Echo Time
TI	= Inversion Time
TIRM	= Turbo Inversion Recovery Magnitude
TR	= Relaxation Time
Tra	= transverse
TSE	= Turbo Spin Echo
