# Supplementary material for: Reduction of claustrophobia during magnetic resonance imaging: methods and design of the "CLAUSTRO" randomized controlled trial
Source: BMC Med Imaging. 2011 Feb 10;11:4. doi: 10.1186/1471-2342-11-4 (PMC3045881; doi:10.1186/1471-2342-11-4)
Supplement: Additional file 4 — Appendix Table S4. Further information on shoulder MR imaging sequences used. [file 1471-2342-11-4-S4.PDF]

Appendix Table 4. Shoulder MR imaging sequences

	Magnetom Avanto	Panorama
**Basic Sequences**		
Generic sequence name	T1w axial	
Vendor sequence name	T1 tra	T1w TSE
TR (ms)	754	932
TE (ms)	14	14
Slices	19	19
Slice thickness (mm)	3.5	3.5
Resulting voxel size (mm)	0.9 x 0.7 x 3.5	0.7 x 0.9 x 3.5
Averages	1	3
Turbo factor	7	7
Acquisition time (min:sec)	2:17	2:47
Generic sequence name	T2w axial	
Vendor sequence name	T2 me2D tra	3D mFFE WATS
TR (ms)	736	1196
TE (ms)	20	13,8
Slices	19	19
Slice thickness (mm)	3.5	3.5
Resulting voxel size (mm)	0.7 x 1.0 x 3.5	0.7 x 1.0 x 3.5
Averages	1	2
Acquisition time (min:sec)	3:19	7:11
Generic sequence name	TIRM coronal	
Vendor sequence name	T1 TIRM cor	STIR
TR (ms)	5700	3000
TE (ms)	28	25
TI (ms)	160	135
Slices	17	17
Slice thickness (mm)	4.0	4.0
Resulting voxel size (mm)	0.6 x 0.8 x 4.0	0.6 x 0.9 x 4.0
Averages	2	4
Turbo factor	12	12
Acquisition time (min:sec)	5:15	6:36
Generic sequence name	PD+T2w TSE coronal	
Vendor sequence name	PD+T2 TSE cor	Dual dr TSE
TR (ms)	2600	2626
First TE (ms)	9.6	13
Second TE (ms)	106	100
Slices	17	17
Slice thickness (mm)	4.0	4.0
Resulting voxel size (mm)	0.6 x 0.8 x 4.0	0.6 x 0.8 x 4.0
Averages	1	3
Turbo factor	10	10
Acquisition time (min:sec)	4:16	5:59
Generic sequence name	T2w TSE parasagittal	
Vendor sequence name	T2 TSE rst fs parasag	T2 TSE SPIR
TR (ms)	5680	4788
TE (ms)	63	63
Slices	19	19
Slice thickness (mm)	4.0	4.0
Resulting voxel size (mm)	0.7 x 0.9 x 4.0	0.7 x 0.9 x 4.0
Averages	2	6
Turbo factor	16	16
Acquisition time (min:sec)	4:04	4:52

Abbreviations:

Cor = coronal
Dual = Double echo
FFE = Fast Field Echo
Me = medic
Parasag = parasagittal
PD = Proton Density
Sag = sagittal
SPIR = Spectral Presaturation Inversion Recovery
STIR = Short T1 Inversion Recovery
T1w = T1-weighted
T2w = T2-weighted
TE = Echo Time
TI = Inversion Time
TIRM = Turbo Inversion Recovery Magnitude
TR = Relaxation Time
Tra = transverse
TSE = Turbo Spin Echo
WATS = WAter only Selection
