# Supplementary material for: Reduction of claustrophobia during magnetic resonance imaging: methods and design of the "CLAUSTRO" randomized controlled trial
Source: BMC Med Imaging. 2011 Feb 10;11:4. doi: 10.1186/1471-2342-11-4 (PMC3045881; doi:10.1186/1471-2342-11-4)
Supplement: Additional file 5 — Appendix Figure S1. MR image analysis form for quantitative and qualitative analysis of head imaging. [file 1471-2342-11-4-S5.PDF]

Appendix Figure 1. MR image analysis form for quantitative and qualitative analysis of head imaging

Reader: **Patient number:**

Analysis time: Start: **End:** **Anatomical region:** Head

Quantitative analysis

(signal intensities (SI) of regions of interest (ROI*): mean value (MV) and standard deviation (SD))

Head imaging		T1 ax MV/SD	T2 ax MV/SD	PD ax MV/SD	TIRM (dark fluid) - ax MV/SD
Air	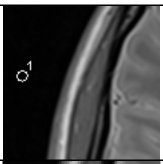	/	/	/	/
Fat tissue	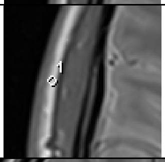	/	/	/	/
Corticospinal fluid	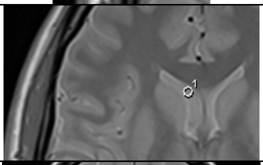	/	/	/	/
Gray brain matter (basal ganglia, caudate nucleus) (1)	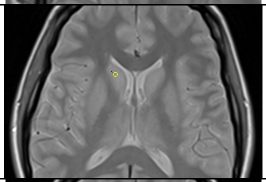	/	/	/	/
White brain matter (corpus callosum, genu) (2)	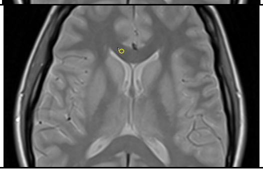	/	/	/	/

Contour clarity index:
Corticospinal fluid (lateral ventricle) – Caudate nucleus – Corpus callosum (genu)

(*) Circular ROI, area of 0.02 – 0.4 cm²

Qualitative analysis

(1 = optimal, 2 = good, 3 = moderate, 4 = poor, 5 = non diagnostic)

Head imaging	T1 ax	T2 ax	PD ax	TIRM ax
Contrast				
Contour clarity				
Image quality				

(1 = none, 2 = minimal, 3 = moderate, 4 = major, 5 = non-diagnostic)

Head imaging	T1 ax	T2 ax	PD ax	TIRM ax
Artifacts				
Noise				

Artifacts caused by: o Motion o Pulsation o Metal o Noise o Other
