# Supplementary material for: Reduction of claustrophobia during magnetic resonance imaging: methods and design of the "CLAUSTRO" randomized controlled trial
Source: BMC Med Imaging. 2011 Feb 10;11:4. doi: 10.1186/1471-2342-11-4 (PMC3045881; doi:10.1186/1471-2342-11-4)
Supplement: Additional file 6 — Appendix Figure S2. MR image analysis form for quantitative and qualitative analysis of spine imaging. [file 1471-2342-11-4-S6.PDF]

Appendix Figure 2. MR image analysis form for quantitative and qualitative analysis of spine imaging

Reader: **Patient number:**

Analysis time: Start: **End:** **Anatomical region:** o CS o TS o LS

Quantitative analysis

(signal intensities (SI) of regions of interest (ROI*): mean value (MV) and standard deviation (SD))

Acquisition: corresponding to mean axial slice: Cervical spine – CVB (standard 4) ____; Thoracic spine – TVB ____; Lumbar spine – LVB ____

Spine imaging		T1 sag MV/SD	T2 sag MV/SD		T2 ax MV/SD height:___
Air	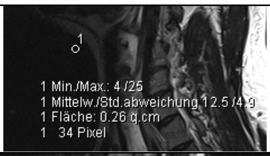	/	/	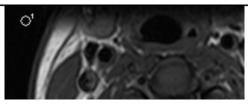	/
Fat tissue	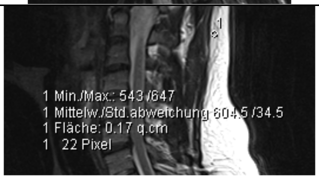	/	/	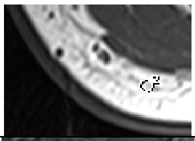	/
(autochthonous) Muscle	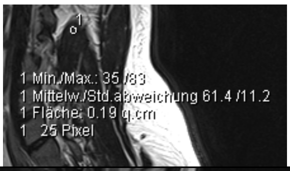	/	/	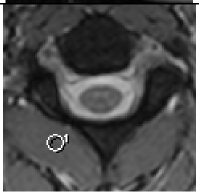	/
Corticospinal fluid	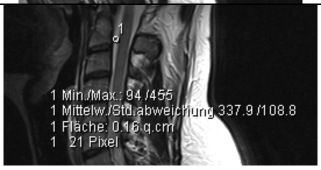	/	/	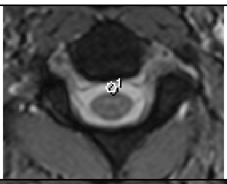	/
Vertebral body	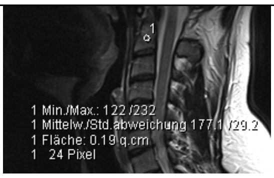	/	/	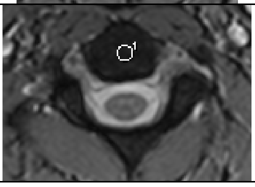	/
Spinal cord	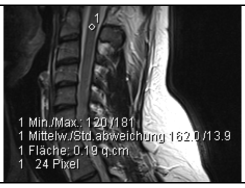	/	/	X	X
Gray matter of spinal cord (1) (not for LS)	X	X	X	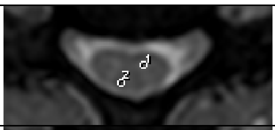	/
White matter of spinal cord (2) (not for LS)	X	X	X	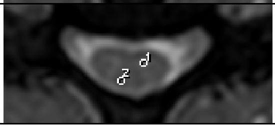	/

Contour clarity index: Gray matter - White matter - Corticospinal fluid - Vertebral body

(*) Circular ROI, area of 0.02 – 0-04 cm²

Qualitative analysis

(1 = optimal, 2 = good, 3 = moderate, 4 = poor, 5 = non-diagnostic)

Spine imaging	T2 sag	T1 sag	T2 ax
Contrast			
Contour clarity			
Image quality			

(1 = none, 2 = minimal, 3 = moderate, 4 = major, 5 = non-diagnostic)

Spine imaging	T2 sag	T1 sag	T2 ax
Artifacts			
Noise			

Artifacts caused by: o Motion o Pulsation o Metal o Noise o Other
