# Supplementary material for: Reduction of claustrophobia during magnetic resonance imaging: methods and design of the "CLAUSTRO" randomized controlled trial
Source: BMC Med Imaging. 2011 Feb 10;11:4. doi: 10.1186/1471-2342-11-4 (PMC3045881; doi:10.1186/1471-2342-11-4)
Supplement: Additional file 7 — Appendix Figure S3. MR imaging analysis form for quantitative and qualitative analysis of shoulder imaging. [file 1471-2342-11-4-S7.PDF]

Appendix Figure 3. MR image analysis form for quantitative and qualitative analysis of shoulder imaging

Reader: **Patient number:**

Analysis time: Start: **End:** **Anatomical region:** Shoulder

Qualitative analysis

(signal intensities (SI) of regions of interest (ROI*): mean value (MV) and standard deviation (SD))

Shoulder imaging		T1 ax MV/SD	Medic ax MV/SD		PD-T2 cor MV/SD	PD cor MV/SD	TIRM cor MV/SD
Air	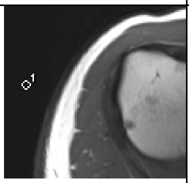	/	/	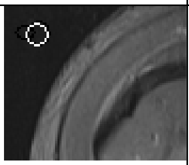	/	/	/
Fat tissue	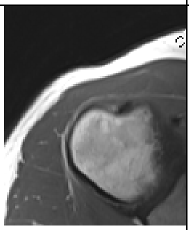	/	/	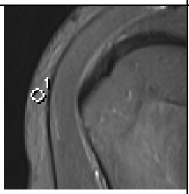	/	/	/
Muscle (deltoid muscle)	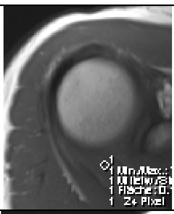	/	/	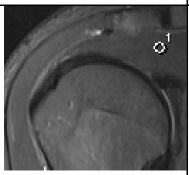	/	/	/
Bone marrow (humerous head)	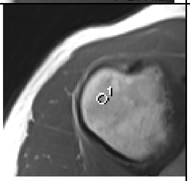	/	/	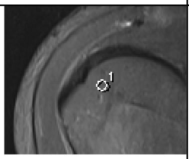	/	/	/
Ligament (axial of biceps muscle, cor/sag of supraspinatus muscle)	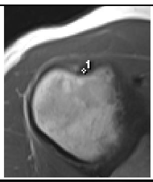	/	/	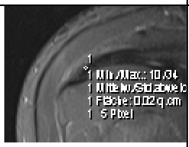	/	/	/
Glenoid labrum	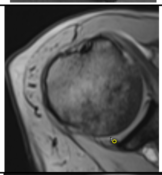	X	/	X	X	X	X
Joint fluid	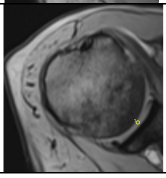	X	/	X	X	X	X

Shoulder imaging		T2 parasag MV/SD
Air	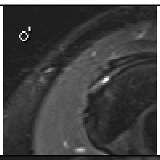	/
Fat tissue	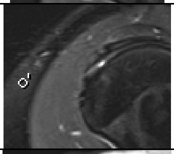	/
Muscle (deltoid muscle)	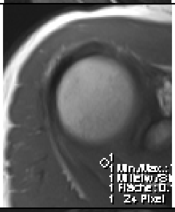	/
Bone marrow (humerus head)	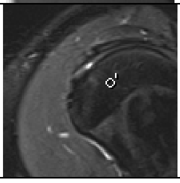	/

Contour clarity index:
Axial: Glenoid labrum – Joint fluid
Coronal: (from lateral imaging) Fat tissue – Muscle (deltoid) – Humerus head

(*) Circular ROI, area of 0.02 – 0.4 cm²

Qualitative analysis

(1 = optimal, 2 = good, 3 = moderate, 4 = poor, 5 = non-diagnostic)

Shoulder imaging	T1 ax	Medic ax	T2 parasag	PD cor	PD-T2 cor	TIRM cor
Contrast						
Contour clarity						
Image quality						

(1 = none, 2 = minimal, 3 = moderate, 4 = major, 5 = non-diagnostic)

Shoulder imaging	T1 ax	Medic ax	T2 parasag	PD cor	PD-T2 cor	TIRM cor
Artifacts						
Noise						

Artifacts caused by: o Motion o Pulsation o Metal o Noise o Other

<u>Diagnostic assessment of rotator cuff muscles/ligaments</u>

(1 = optimal, 2 = good, 3 = moderate, 4 = poor, 5 = non-diagnostic)

Rotator cuff		T1 ax	Medic ax		PD-T2 cor	PD cor	TIRM cor
Supraspinatus muscle	X	X	X	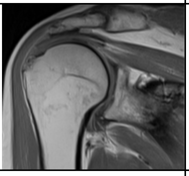			
Subscapularis muscle	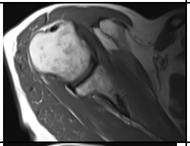			X	X	X	X
Infraspinatus muscle	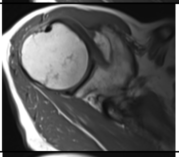			X	X	X	X
Teres minor muscle	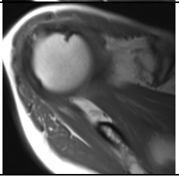			X	X	X	X

Glenoid labrum

(1 = optimal, 2 = good, 3 = moderate, 4 = poor, 5 = non-diagnostic)

Glenoid labrum		T1 ax	Medic ax
Anterior	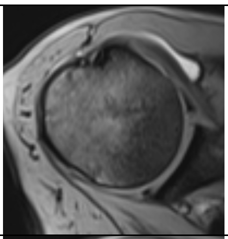		
Posterior	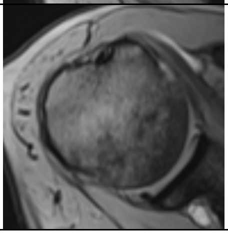		

Graduation of fatty infiltration of rotator cuff muscles according to Goutallier [31]:

0	No fat
1	A few streaks of fat
2	More muscle than fat
3	As much muscle as fat
4	More fat than muscle

Graduation of muscle atrophy according to Warner [32]:

1	None
2	Mild
3	Moderate
4	Severe
